# Editorial: Noncanonical functions of Aminoacyl-tRNA synthetases

**DOI:** 10.3389/fphys.2023.1165515

**Published:** 2023-02-23

**Authors:** Litao Sun, Xiao-Long Zhou, Zhong-Wei Zhou, Haissi Cui

**Affiliations:** ^1^ School of Public Health, Sun Yat-sen University, Shenzhen, China; ^2^ State Key Laboratory of Molecular Biology, CAS Center for Excellence in Molecular Cell Science, Shanghai Institute of Biochemistry and Cell Biology, Chinese Academy of Sciences, University of Chinese Academy of Sciences, Shanghai, China; ^3^ School of Medicine, Sun Yat-sen University, Shenzhen, China; ^4^ Department of Chemistry, University of Toronto, Toronto, ON, Canada

**Keywords:** aminoacyl-tRNA synthetases, non-canonical functions, genetic diseases, genomic duplication, cysteine incorporation, virus

Aminoacyl-tRNA synthetases (aaRS) catalyze the first step of protein synthesis by attaching amino acids to tRNAs. During this key step, aminoacyl-tRNA synthetases further ensure the correct interpretation of the genetic code by providing the ribosome with the building blocks for proteins (Woese et al., 2000). However, this well-studied and strictly conserved “canonical” function of aaRS does not encompass the whole complexity of this ancient protein family. During evolution, aaRS have been co-opted, copied, extended, and mutated to fulfill additional functions (Guo and Schimmel, 2013; Sun et al., 2016; Zhang et al., 2021). In addition, alternatives to aaRS have also been found, as prokaryotes often do not contain a full set of aaRS for all the 20 proteinogenic amino acids that are shared between all living beings.

In this issue, the non-canonical functions of aaRS are discussed and showcased across different kingdoms. In addition to the non-canonical use of aaRS, alternative pathways of tRNA aminoacylation are also discussed. Together, they paint a picture of the versatility of aaRS and the creativity of nature in making use of them.

Mukai et al. demonstrate alternative pathways to encode cysteine in prokaryotes and find that they are far more widespread than previously assumed. Cysteine incorporation can occur as a two-step mechanism, which has also been described for other amino acids. However, the aminoacylation of tRNAs with, for example, glutamine, relies on the relaxed specificity of the aaRSs which recognizes a chemically very similar amino acid, glutamate. The prokaryote thereby effectively “saves” the addition of a designated aaRS. Cysteine incorporation on the other hand requires a specialized aaRSs, which attaches a phosphoserine to tRNA, which is then modified to a cysteine (Mukai et al., 2021). Mukai et al. explore how common this mode is by using bioinformatical techniques and gain surprising insights into the creativity of interpreting a shared genetic code across species.

In contrast, Krahn et al. take the opposite direction. Instead of looking into different ways in which the same amino acid can be attached to a tRNA, they explore how to get the most use out of aaRS, which is by copying them (Krahn et al., 2022). Discussing the most extreme form of co-opting, which is to make a copy and use it for an alternative function, they explore the potential of aaRS-like proteins in alternative functions (or even in the same). AaRS are mostly thought of as single-copy genes to keep evolutionary pressure high, as misacylation could have fatal consequences due to protein misfolding and a loss of function if key residues are exchanged. Despite this, gene duplications have been found, and in an excellent summary, Krahn et al. discuss why they persist: while some have increased functionality under extreme conditions, enabling accurate tRNA aminoacylation where the primary enzyme fails, others lose or adapt their aminoacylation activity and instead confer cell-signaling functions or modifications of other biomolecules (Krahn et al., 2022). The appearance of these duplicated and co-opted aaRS across all kingdoms impressively demonstrates the potential role of aaRS in a variety of functions.

In humans, this diversity of function especially comes into focus during disease states. Viral infections have tremendous societal impact, as demonstrated by the COVID-19 pandemic. Interestingly, Feng et al. found that several aaRS interact with SARS-CoV-2 proteins and showcase their distinct regulation and post-translational modification upon infection, supporting the hypothesis that they might play key roles during viral infections (Feng et al., 2021). Exploring this angle of aaRS biology holds tremendous therapeutic potential, as individual aaRS domains can hold the key to modulate disease progression, as demonstrated in recent clinical studies. By taking a deep dive into the interplay with virus-derived biomolecules, the stage is set for a reimagining of the roles that aaRS hold.

Another consideration regarding aaRS and human health is that genetic mutations in aaRS can cause disease in patients. Turvey et al. discuss mono and biallelic mutations that lead to neurodevelopmental and degenerative diseases in both cytoplasmic and mitochondrial aaRS as well as autoimmune disorders caused by antibodies raised against endogenous aaRS (Turvey et al., 2022). This impressive overview contains a list of known disease-causing mutations in humans, which will surely be a resource for future studies. Thanks to the hard work and critical review by the authors, this article provides a wealth of reference material and summarizes and contextualizes recent findings in the field.
